# Dissecting the molecular control of immune cell accumulation in the inflamed joint

**DOI:** 10.1172/jci.insight.151281

**Published:** 2022-04-08

**Authors:** Catriona T. Prendergast, Robert A. Benson, Hannah E. Scales, Caio Santos Bonilha, John J. Cole, Iain McInnes, James M. Brewer, Paul Garside

**Affiliations:** Institute of Infection, Immunity and Inflammation, College of Medical, Veterinary and Life Sciences, University of Glasgow, Glasgow, United Kingdom.

**Keywords:** Autoimmunity, Inflammation, Arthritis, Autoimmune diseases, Cell migration/adhesion

## Abstract

Mechanisms governing entry and exit of immune cells into and out of inflamed joints remain poorly understood. We sought herein to identify the key molecular pathways regulating such migration. Using murine models of inflammation in conjunction with mice expressing a photoconvertible fluorescent protein, we characterized the migration of cells from joints to draining lymph nodes and performed RNA-Seq analysis on isolated cells, identifying genes associated with migration and retention. We further refined the gene list to those specific for joint inflammation. RNA-Seq data revealed pathways and genes previously highlighted as characteristic of rheumatoid arthritis in patient studies, validating the methodology. Focusing on pathways associated with cell migration, adhesion, and movement, we identified genes involved in the retention of immune cells in the inflamed joint, namely junctional adhesion molecule A (JAM-A), and identified a role for such molecules in T cell differentiation in vivo. Thus, using a combination of cell-tracking approaches and murine models of inflammatory arthritis, we identified genes, pathways, and anatomically specific tissue signatures regulating cell migration in a variety of inflamed sites. This skin- and joint-specific data set will be an invaluable resource for the identification of therapeutic targets for arthritis and other inflammatory disorders.

## Graphical abstract

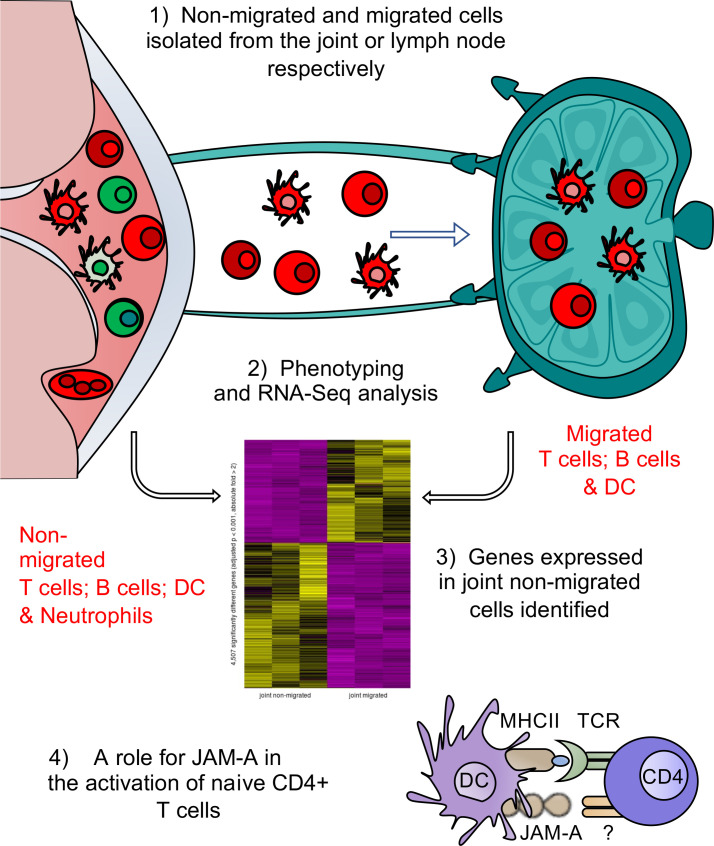

## Introduction

Rheumatoid arthritis (RA) is a chronic autoimmune disease that is characterized by joint and synovial swelling and the infiltration and accumulation of inflammatory cells in the normally sparsely populated joint space (reviewed in McInnes and Schett; ref. 1). This cellular accumulation in the inflamed joint can occur through increased recruitment, survival, and/or proliferation of cells in the articular environment. Equally, accumulation could occur through a failure in cellular egress from the inflamed joint. Numerous cell types are found within the inflamed joint environment, which play important roles in directly causing joint pathology and maintaining the underlying autoimmune responses (1).

The development of autoimmune responses in RA is thought to occur in the lymphoid organs (lymphoid phase). The direct cause of this breach of self-tolerance is unknown but involves a number of environmental/lifestyle risk factors, and genome-wide association studies have identified polymorphisms in genes related to T cell activation as risk factors (i.e., HLA-DR4 and PTPN22). After this breach of tolerance, inflammatory cells, including T cells, neutrophils, and macrophages, are recruited to the joints (articular phase). The molecular pathways that promote the recruitment of these cells are well characterized, involving a variety of cytokines, chemokines, adhesion molecules, and their receptors (2, 3). For example, it has been shown that blocking the chemokine receptor CXCR3 inhibits T cell recruitment to inflamed joints (4), while CCR2 is known to be essential for the recruitment of monocytes to tissues. Less is understood about the molecules responsible for either retaining cells within tissues or mediating their egress. Retention of T cells may be dependent on adhesion molecules directly and/or the presence of cognate antigen and interaction with antigen presenting cells (APCs), such as DCs and antigen-specific B cells. Indeed, we have previously demonstrated in a murine model of breach of self-tolerance that T cells can interact with DCs in the inflamed joint in a fashion consistent with the cognate interactions routinely observed in lymph nodes (LNs) during antigen presentation (5). Less is known about the molecular pathways that govern the egress of cells from inflamed joints. Pharmacological inhibition of the sphingosine-1/phosphate (S1P) pathway has been shown not only to prevent lymphocyte egress from LNs (6, 7) but also to inhibit DC migration from joints to LNs (8, 9).

In order to further our understanding of the genes and molecules governing cellular egress from and retention in inflamed joints, we utilized a murine model of inflammatory arthritis (10) and a transgenic mouse expressing the photoconvertible protein Kaede (11). Using these models, we identified cells that had migrated out of inflamed joints to the draining LNs and those that were retained in the inflamed articular tissues. Comparison of these 2 leukocyte populations generated a data set of genes specific to the movement of inflammatory cells out of the joint. Genes common to general inflammation were excluded through the inclusion of corresponding cell populations derived from an inflamed nonarticular tissue, in this case the skin. In this way, an invaluable data set was generated. Data mining using pathway analysis identified several genes of interest to take forward for further exploration. Initial studies demonstrated that these provide targets of therapeutic interest.

## Results

### Identification of cellular egress from inflamed joints to draining LNs.

Considerable efforts have focused on pathways recruiting cells to the inflamed joint; however, there has been little focus on mechanisms regulating their exit. We therefore took advantage of transgenic mice expressing the photoconvertible protein Kaede to optically tag cells in murine joints during inflammatory arthritis and determine the extent to which cellular egress occurred. In this system, UV conversion of Kaede green to red irreversibly identifies cells present in the joint at the time of photoconversion (11). Cells that are subsequently retained in the joint are identifiable as Kaede red^+^, and any Kaede red^+^ cells present in the popliteal LNs (pLNs) are known to have migrated from the photoconverted joint.

**Figure 1:**
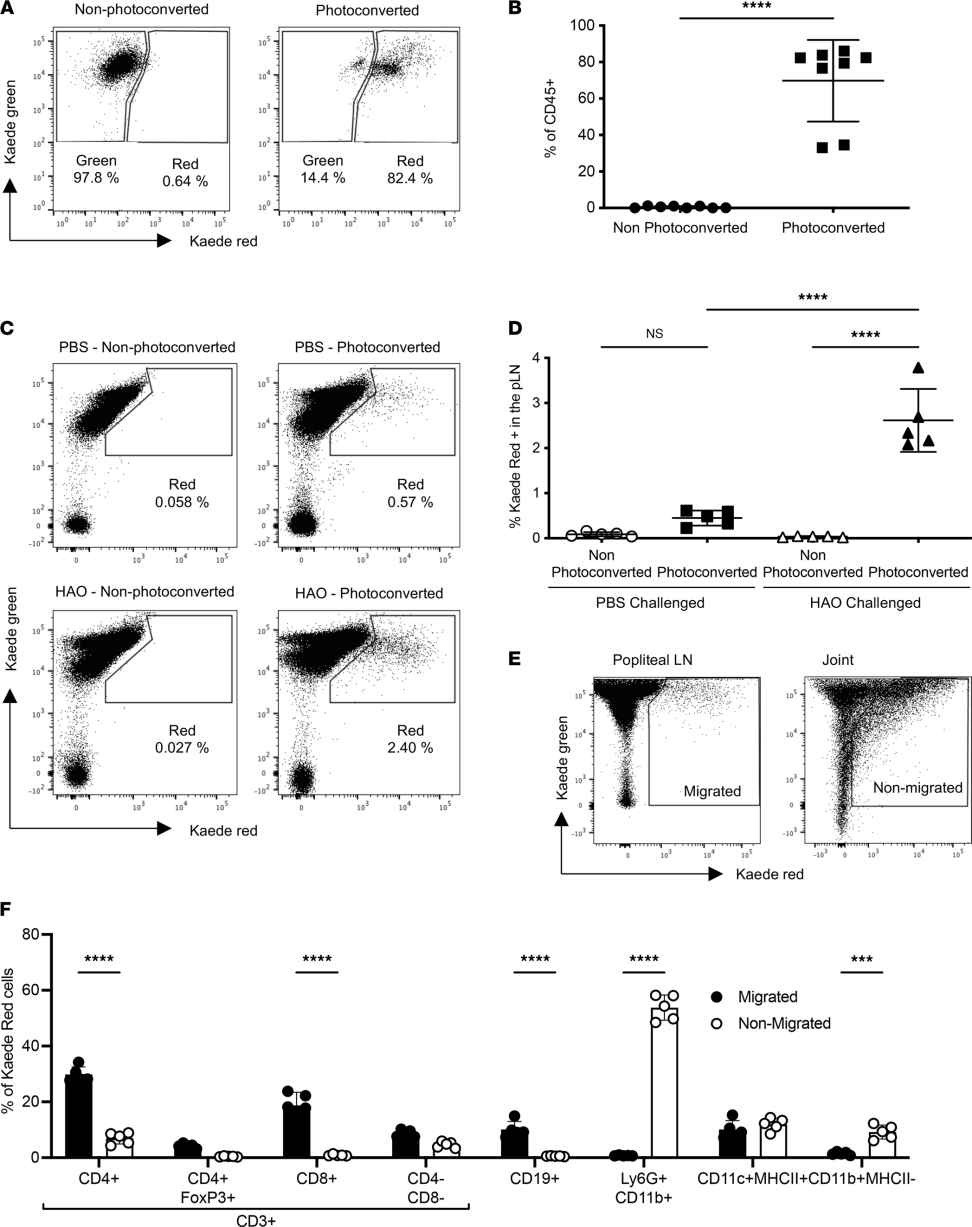
After HAO challenge, a significant population of cells migrate out of the inflamed joint to the popliteal lymph nodes. Inflammatory arthritis was induced in female Kaede recipient mice; inflamed joints were photoconverted by exposure to UV light for a total of 30 seconds on day 3 after HAO challenge. (**A**) Representative flow cytometry plots showing CD45^+^ cells recovered from inflamed joints highlighting Kaede green versus red in non-photoconverted and photoconverted HAO-challenged mice euthanized immediately after photoconversion. (**B**) Quantitative analysis of extent of photoconversion showing the percentage of Kaede red^+^ cells. Statistical differences between the treatment groups were determined using unpaired 2-tailed *t* test; *n* = 8. (**C**) Representative flow cytometry plots showing Kaede green versus red in CD45^+^ cells recovered from the popliteal lymph nodes (pLNs) of PBS- or HAO-challenged mice at 24 hours after photoconversion. Cells were previously gated on lymphocytes, single cells, viable cells, and CD45^+^ cells. (**D**) Quantitative analysis of the percentage of Kaede red^+^ cells in the pLNs from not photoconverted or photoconverted mice after PBS or HAO challenge. Statistical differences determined using 1-way ANOVA with Tukey’s multiple-comparison test; *n* = 5. (**E**) Representative flow cytometry plots showing Kaede green versus red in the pLNs and joints of HAO-challenged mice using the OVA-RA model. Gates represent the population of Kaede red^+^ cells that were later sorted via FACS for RNA-Seq analysis. (**F**) Comparison of the cellular composition of the 2 Kaede red^+^ populations: CD45^+^Kaede red^+^ migrated cells from the inflamed joint and CD45^+^Kaede red^+^ nonmigrated cells still within the inflamed joint. Statistical differences between the treatment groups were determined using 2-way ANOVA with Holm-Šídák multiple-comparison test; *n* = 5. Data are representative of 3 separate experiments; each symbol represents an individual animal; mean ± SD shown. ***P < 0.001, ****P < 0.0001. HAO, heat-aggregated ovalbumin.

Articular inflammation was induced in Kaede mice using a previously described model of inflammatory arthritis (Supplemental Figure 1A; supplemental material available online with this article; https://doi.org/10.1172/jci.insight.151281DS1) (10), and joint tissues were photoconverted on day 3 after induction of articular inflammation. Flow cytometry of joint tissues immediately after UV exposure confirmed successful photoconversion of Kaede green to red (~70% of the CD45^+^ cells photoconverted to Kaede red) (Figure 1, A and B) and only a slight increase in the percentage of Kaede red^+^ cells in the draining LNs compared with the unswitched control (Supplemental Figure 1, B and C). The proportion of Kaede red^+^CD45^+^ cells in the joint decreased 24 hours after photoconversion (data not shown), probably reflecting continuing recruitment of nonconverted Kaede green cells from the periphery. Importantly, at 24 hours after photoconversion, Kaede red^+^ cells were detected in the joint-draining pLNs, with a significant increase in cells migrating from inflamed versus noninflamed joints (2.1% to 3.7% versus 0.2% to 0.6% of the CD45^+^ cells, respectively) (Figure 1, C and D).

To determine which cells were remaining within the inflamed joint and those migrating out, we compared the phenotype of Kaede red^+^ cells in the joint (nonmigratory) and those in the pLN (migratory) via flow cytometry (Figure 1, E and F). Comparison between the 2 populations revealed that the migratory population contained a significantly greater proportion of T cells (CD3^+^CD4^+^ and CD3^+^CD8^+^) and B cells (Figure 1F). By contrast, the percentages of CD11b^+^Ly6G^+^ neutrophils and CD11b^+^MHCII^–^ (monocytes) were higher in the nonmigratory compared with migrated leukocytes (Figure 1F). Comparable proportions of DCs, Tregs (CD3^+^CD4^+^FoxP3^+^), and nonconventional T cells (CD3^+^CD4^–^CD8^–^) were found in the 2 populations (Figure 1F). Analysis using t-distributed stochastic neighbor embedding (t-SNE) allowed the visualization of the different populations using 2 flow cytometry panels (Supplemental Figure 2, A and B), demonstrating that the migratory and nonmigratory populations were composed of distinct cell types.

### Migratory and nonmigratory cells display distinct transcriptional profiles.

**Figure 2:**
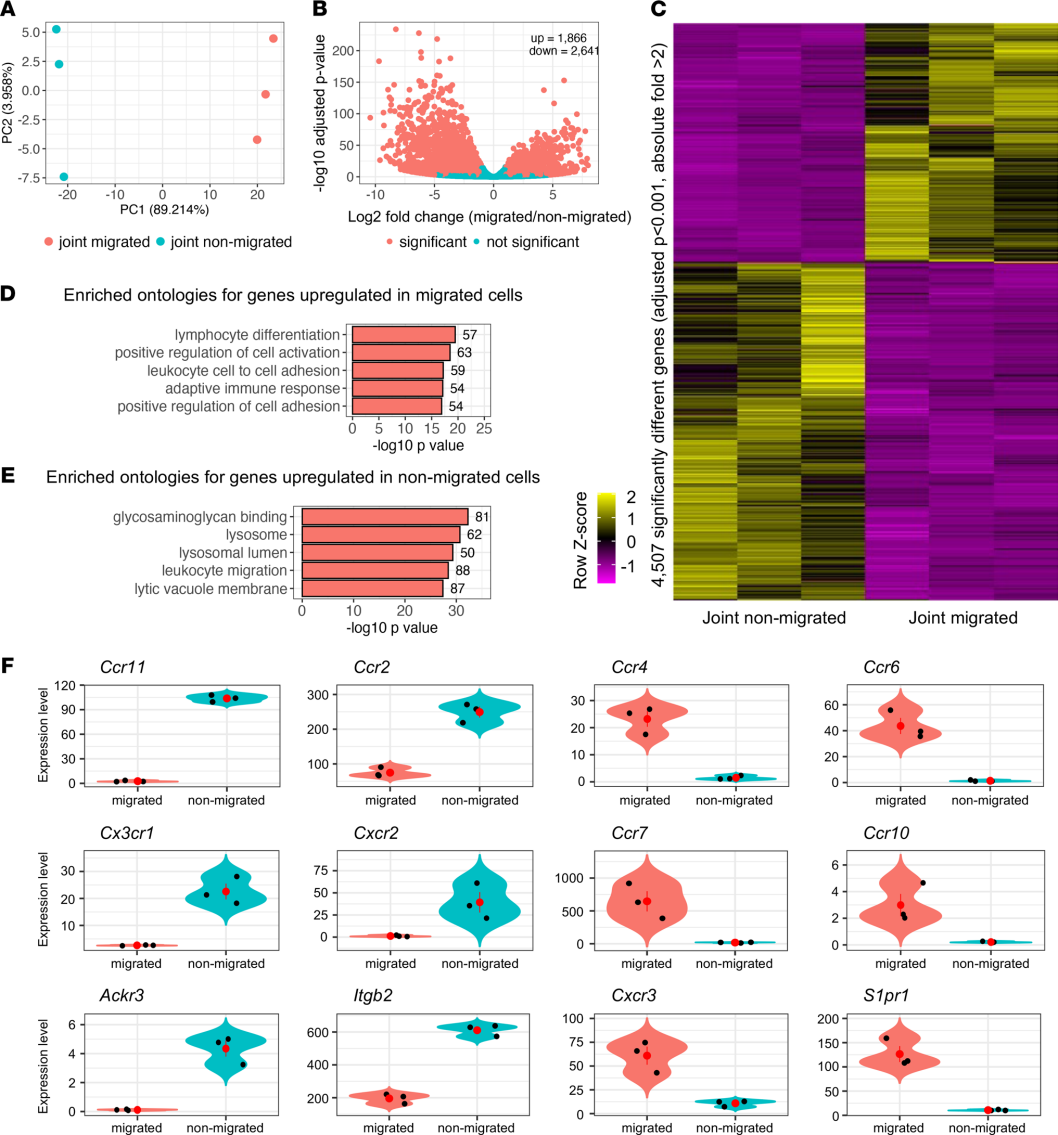
Analysis of differentially expressed genes between the joint migrated and the joint nonmigrated cells via RNA-Seq. RNA-Seq was performed on Kaede red^+^ cells FACS sorted from the inflamed joints and from the draining popliteal LNs. Three biological replicates were obtained for each group, taken from pooled groups of 8–18 matched male and female mice. (**A**) Principal component analysis (PCA) of joint migrated versus nonmigrated cells. (**B**) Volcano plot highlighting genes that were more than 2-fold upregulated or downregulated between the joint migrated versus the joint nonmigrated populations (1866 genes upregulated; 2641 genes downregulated). (**C**) Heatmap showing the 4507 genes that were significantly differentially regulated genes between the 2 populations. (**D**) Pathway analysis highlighting the enriched ontologies in the genes upregulated in the migrated cells and (**E**) the enriched ontologies in the genes upregulated in the nonmigrated cells (DAVID software). The number indicates how many genes in the data set are within each specific ontology. (**F**) Violin plots highlighting the differential expression of specific genes within the data set. Orange represents the migrated and blue represents the nonmigrated population; black dots represent individual replicates and the red dot represents the mean ± SD.

To understand the molecular cues governing the egress of cells from the inflamed joint, the migratory and nonmigratory Kaede red^+^ cells were sorted by FACS from pooled samples of either the pLN or joint, respectively, in triplicate, at day 4 after challenge (24 hours after photoconversion). RNA-Seq was performed on the Kaede red^+^ cells obtained from the pLN (joint migratory) and joint (joint nonmigratory), and the differential expression of genes is shown in Supplemental Table 1. Principal component analysis revealed the similarity between the biological replicates as well as the differences between the 2 groups (Figure 2A). We identified 4507 differentially expressed genes, with 1866 genes more highly expressed by migratory cells and 2641 by nonmigratory cells (Figure 2, B and C). Network analysis of the upregulated genes in the nonmigrated cells suggested involvement of a variety of pathways related to immune activation, cytokine signaling, and metabolism (Supplemental Figure 3). Pathway analysis of the genes more highly expressed by the migratory cells revealed enrichment of genes associated with cell adhesion and activation of adaptive immune responses (Figure 2D and Supplemental Table 2). Cells remaining in the joint (nonmigratory) had elevated expression levels of genes relating to binding to glycosaminoglycans and leukocyte migration (Figure 2E and Supplemental Table 3). Closer examination of differentially expressed genes relating to cellular migration in RA and associated mouse models demonstrated expression patterns consistent with the published literature (Figure 2F). Higher levels of *Ccr1*, *Ccr2*, *Cxcr2*, *Cx3cr1*, *Ackr3*, and *Atgb2* were found in the nonmigratory cells in the inflamed joint, and migratory CD45^+^ cells in the pLN expressed elevated levels of *Ccr4*, *Ccr6*, *Ccr7*, *Ccr10*, *Cxcr3*, and *S1pr1* (Figure 2F).

### CD4^+^ T cell egress from inflamed joints is regulated by S1P responsiveness.

In order to further validate our methodology, we selected one of the genes, *S1pr1*, to further evaluate its role in the migration of cells out of the joint. S1P and its receptor S1P1 have already been shown to be important in several diseases and are of therapeutic interest in several autoimmune settings (12), and compounds that inhibit S1P1 signaling and function in vivo are readily available. Because cells that had migrated from inflamed joints were seen to express higher levels of *S1pr1*, we hypothesized that responsiveness to S1P may contribute to migration out of the arthritic joint. As such, we took advantage of fingolimod (FTY720), a functional S1P1 antagonist, to determine its contribution to cell migration out of or retention in articular tissues.

**Figure 3:**
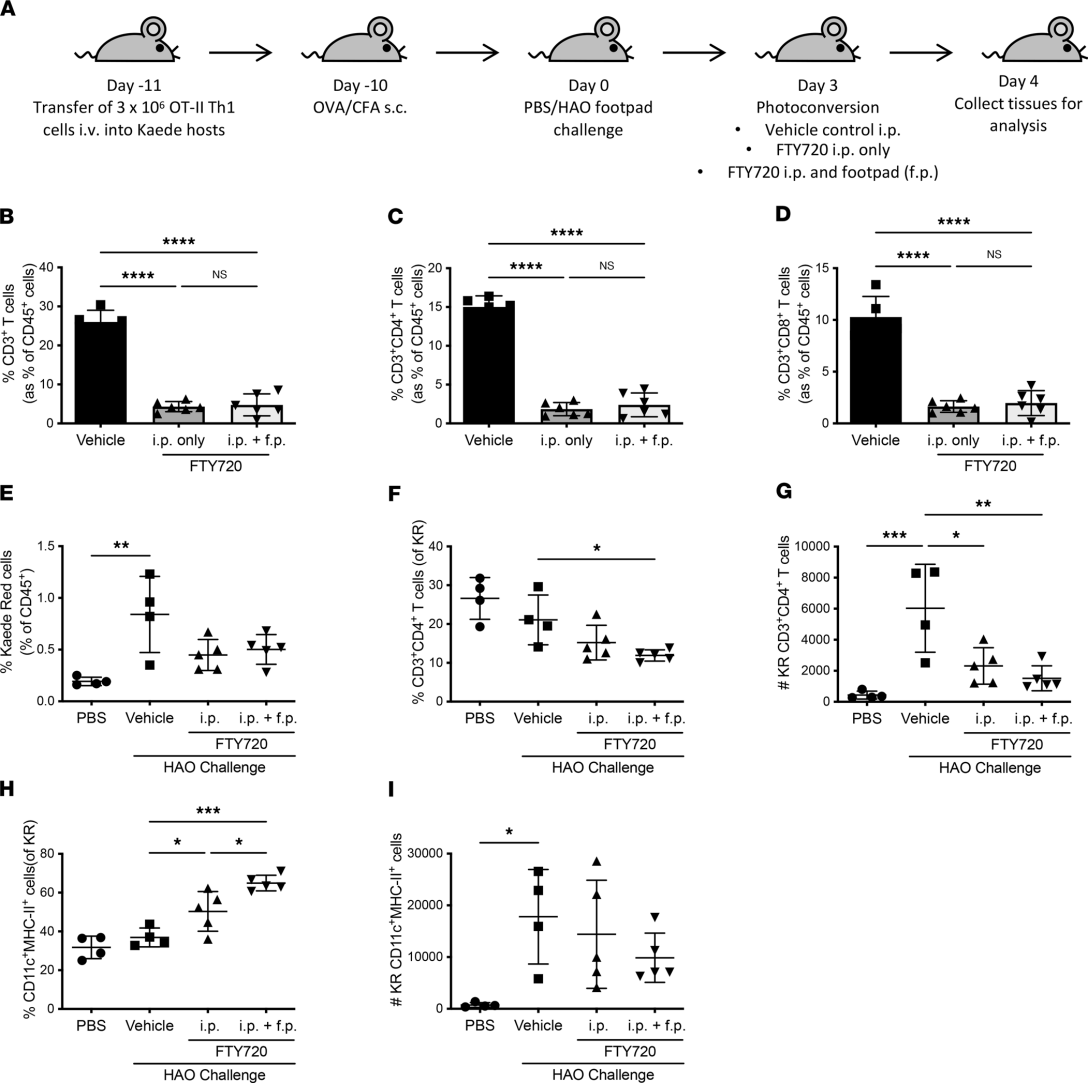
Blockade of the S1P pathway via FTY720 administration affects the movement of cells out of the inflamed joint. (**A**) Schematic showing the OVA-RA model timeline using female Kaede mice as the recipient mice. Mice received either vehicle control i.p. (*n* = 4) or 1 mg/kg FTY720 i.p. (*n* = 5) only or 1 mg/kg FTY720 i.p. in addition to 1 mg/kg FTY720 s.c. directly in the footpad (*n* = 5) on day 3 after HAO challenge; an untreated PBS-challenged group served as a control (*n* = 4). Twenty-four hours later, mice were euthanized and the popliteal lymph nodes collected for analysis via flow cytometry. To determine the efficacy of the FTY720, flow cytometry was used to determine the percentages of (**B**) CD3^+^ T cells (gated on CD45^+^ cells) and, more specifically, of (**C**) CD3^+^CD4^+^ and (**D**) CD3^+^CD8^+^ T cells in the blood after either vehicle or 2 different dosages of FTY720 in all HAO-challenged groups. Statistical differences between the treatment groups were determined using 1-way ANOVA with Tukey’s multiple-comparison test. (**E**) Summary of flow cytometric analysis s showing the percentage of CD45^+^ cells that are Kaede red^+^ migrated cells in the popliteal LNs after PBS or HAO footpad challenge followed by vehicle or FTY720 administration. Flow cytometric analysis of the Kaede red^+^ population specifically shows (**F**) the percentage of the Kaede red^+^ population that were CD3^+^CD4^+^ T cells, (**G**) the absolute number of CD3^+^CD4^+^Kaede red^+^ T cells, (**H**) the percentage of Kaede red^+^ cells that were CD11c^+^MHC-II^+^, and (**I**) the absolute number of CD11c^+^MHC-II^+^Kaede red^+^ cells. Statistical differences between the treatment groups were determined using 1-way ANOVA with Tukey’s multiple-comparison test. Data are representative of 2 separate experiments; each symbol represents an individual animal; mean ± SD shown. *P < 0.05, **P < 0.01, ***P < 0.001, ****P < 0.0001.

Arthritis was induced in Kaede mice as described previously, with FTY720 administered at day 3 after challenge, via either i.p. injection alone or with simultaneous footpad injection to ensure local availability (Figure 3A). Given that FTY720 sequesters circulating lymphocytes in lymphoid tissue, we opted for a single treatment at day 3 after footpad challenge, allowing articular inflammation to establish prior to intervention. Inflamed joints were also photoconverted at this point, and draining LNs were collected 24 hours later to determine the impact on cellular egress (Figure 3A). Loss of T cell populations from the peripheral blood confirmed the activity of FTY720 (Figure 3, B–D). No significant impact was observed on the overall migration of cells out of inflamed joints (Figure 3E) or the retention of cells in inflamed joints (Supplemental Figure 4A). However, CD4^+^ T cell emigration was significantly reduced, with reductions in the percentage and number of CD3^+^CD4^+^Kaede red^+^ cells in the LNs after FTY720 treatment compared with the vehicle control (Figure 3, F and G). In the joint, no differences in the proportion of the retained CD3^+^CD4^+^Kaede red^+^ cells were observed (Supplemental Figure 4B). Additionally, a significantly increased percentage of Kaede red^+^ cells were found to be CD11c^+^MHCII^+^ DCs, probably reflecting the change in T cell proportions, as the number of DCs migrating out of joints was unaffected (Figure 3, H and I). In the joint, the proportion of the retained CD11c^+^MHCII^+^Kaede red^+^ DCs was reduced in the animals treated with both systemic and local FTY720 compared with those treated with systemic FTY720 only (Supplemental Figure 4C).

### Joint migratory and nonmigratory cells display transcriptional profiles unique to articular inflammation.

**Figure 4:**
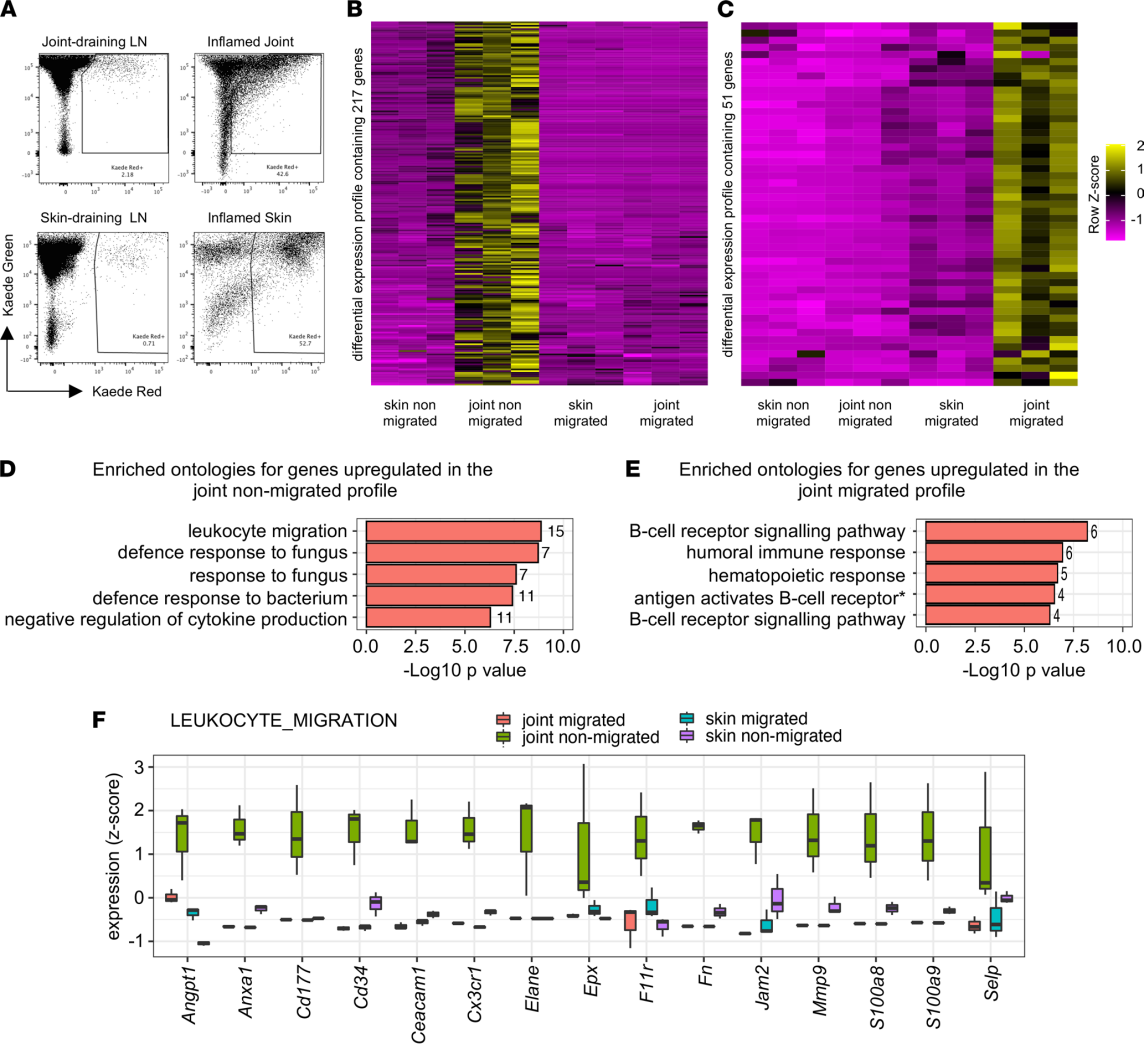
Comparison of a nonspecific inflamed site versus the inflamed joint to identify a joint-specific differentially regulated gene list. (**A**) Representative flow cytometry plots showing the Kaede green versus Kaede red^+^ cells in the joint-draining LNs, inflamed joint, skin-draining LNs, and inflamed skin. Gates represent population of Kaede red^+^ cells sorted via FACS and used for RNA-Seq analysis. Three biological replicates were obtained for each group, taken from pooled groups of 8–18 matched male and female mice. (**B**) Heatmaps highlighting genes that are significantly upregulated in the joint nonmigrated cell population and (**C**) in the joint migrated cell population. (**D**) Pathway analysis highlighting the enriched ontologies for genes in the joint nonmigrated population and (**E**) in the joint migrated population. The numbers in the graphs represent the number of genes in the data set involved in each pathway. (**F**) Box-and-whisker plots showing the relative expression of each of the genes in the “leukocyte migration” pathway in the joint migrated, joint nonmigrated, skin migrated, and skin nonmigrated groups. The box represents the mean, lower and upper hinges correspond to the 25th and 75th percentiles, and the whiskers represent the maximum and minimum.

Many of the genes identified by our transcriptional analysis of migratory versus nonmigratory cells will be common to egress from a variety of inflamed tissues, as evidenced by the differential expression of *S1pr1*, which has been shown to be relevant to several inflammatory settings. To establish which genes were specific to articular inflammation, we generated a second transcriptional data set from migratory and nonmigratory cells from inflamed skin. By employing the same protocol for induction of arthritis but changing the site of challenge to the ear pinnae, we induced a directly comparable skin inflammation. As in our arthritis model, nonmigratory and migratory leukocytes were identified as CD45^+^Kaede red^+^ cells in the inflamed tissue and draining auricular LNs, respectively (Figure 4A). To generate transcriptional profiles for the skin, RNA-Seq was performed on FACS-sorted nonmigratory Kaede red^+^ cells remaining within the skin (skin nonmigrated) and migratory Kaede red^+^ cells from auricular LNs (skin migrated). These 2 populations were then directly compared with the joint nonmigrated (Supplemental Figure 5A) and joint migrated (Supplemental Figure 5B) populations generated previously. Two lists of genes specific to the joint were identified (Supplemental Table 4): 217 genes were upregulated in the nonmigrated population (compared with the other 3 populations) (Figure 4B) and 51 genes upregulated in the migrated population (Figure 4C). Pathway analysis of the genes differentially expressed in the joint nonmigrated cells revealed enrichment for pathways involved in leukocyte migration (Figure 4D and Supplemental Table 5). The genes upregulated in the cells migrating out of the inflamed joint were enriched for pathways involved in B cell signaling and the humoral immune response (Figure 4E and Supplemental Table 6). The upregulated B cell signaling genes were *Cd19*, *Cd22*, *Cd79a*, *Cd79b*, *Cd21*, and *Rasgrp3*, suggesting that there may have been increases in B cell maturation and antigen processing within the migrated cells (Supplemental Figure 4C). The differential expression of genes by migrated and nonmigrated cells, in addition to reflecting the changes required for migration, may also have been the result of adapting to or interacting with a new tissue. We therefore focused on the genes known to be involved in leukocyte migration in the nonmigrated cells in the joint, identifying 15 genes specifically upregulated in joints compared with the other 3 populations (Figure 4F). Given the T cell dependency of both our model and RA, we used gene bank data (Immunological Genome Project, Gene Skyline, Microarray) (13) to determine the expression of these 15 genes by DCs or CD4^+^ T cells in C57BL/6 LNs and spleens. Of the genes, *Anxa1*, *Cd177*, *Ceacam1*, *Cx3xr1*, *F11r*, and *Mmp9* were expressed at higher levels by DCs or CD4^+^ T cells. Because of the preponderance of neutrophils in the nonmigrated population, we also examined expression by neutrophils and identified that *Anxa1* and *Mmp9* were also highly expressed by neutrophils. Of the remaining 4 T cell– and DC-expressed genes, we chose *F11r*, the gene coding for junctional adhesion molecule A (JAM-A), as a target to take forward to further investigate its role in inflammatory arthritis.

### A higher percentage of the cells retained in the inflamed joint are JAM-A^+^ compared with the cells migrated to the LNs.

**Figure 5:**
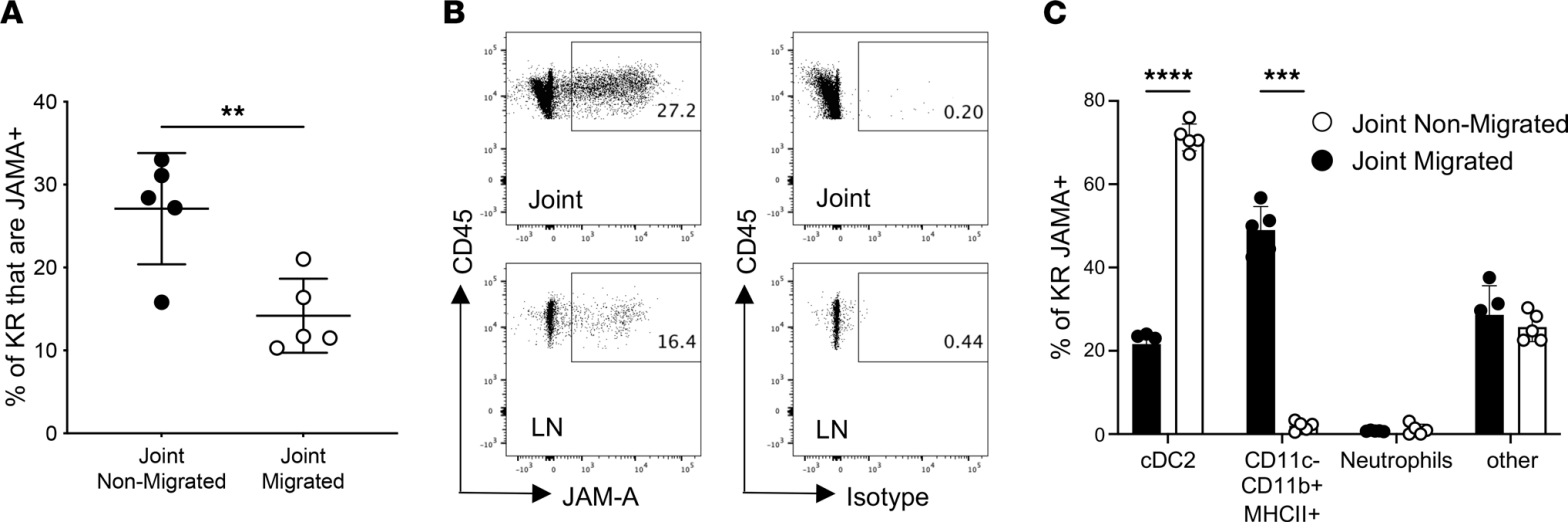
JAM-A is expressed on a higher percentage of the cells retained in the joint. Inflammatory arthritis was induced in female Kaede mice and the inflamed joint photoconverted at day 4 after HAO challenge; tissues were harvested 24 hours later and analyzed by flow cytometry. (**A**) Percentage of the joint nonmigrated and migrated cells (Kaede red+) that expressed JAM-A. (**B**) Representative flow cytometry plots showing the expression of JAM-A (left panel) or the isotype control (right panel) on Kaede red^+^ cells in the joint. (**C**) Bar chart showing the phenotype of the JAM-A^+^Kaede red^+^ cells in the joint nonmigrated and migrated populations. Statistical differences between the treatment groups were determined using 2-way ANOVA with Holm-Šídák multiple-comparison test; *n* = 5. Data are from a single experiment; each symbol represents an individual animal; mean ± SD shown. **P < 0.01, ***P < 0.001, ****P < 0.0001.

In order to confirm the RNA-Seq data showing an increase in JAM-A expression by joint nonmigrated cells compared with migrated cells, arthritis was induced in Kaede mice, and nonmigrated and migrated cells were identified, as shown in Supplemental Figure 6. A significantly higher percentage of the nonmigrated Kaede red^+^ cells in the joint expressed JAM-A than the migrated Kaede red^+^ cells in the pLN (Figure 5, A and B). The phenotype of the Kaede red^+^JAM-A^+^ cells in the joints and LNs was determined by the expression of Ly6G, CD11b, MHCII, CD11c, and CD8. In the joint, the majority of the retained JAM-A^+^ cells were CD11c^–^CD11b^+^MHCII^+^ cells, potentially macrophages or inflammatory monocytes, and around 20% were conventional type 2 DCs (cDC2; CD11c^+^CD11b^+^MHCII^+^), whereas in the draining LNs, the majority of the migrated JAM-A^+^ cells were cDC2 (Figure 5C).

### Blockade of JAM-A in vivo does not alter the development of arthritis but reduces the proliferation and differentiation of naive T cells in response to primary antigen challenge.

**Figure 6:**
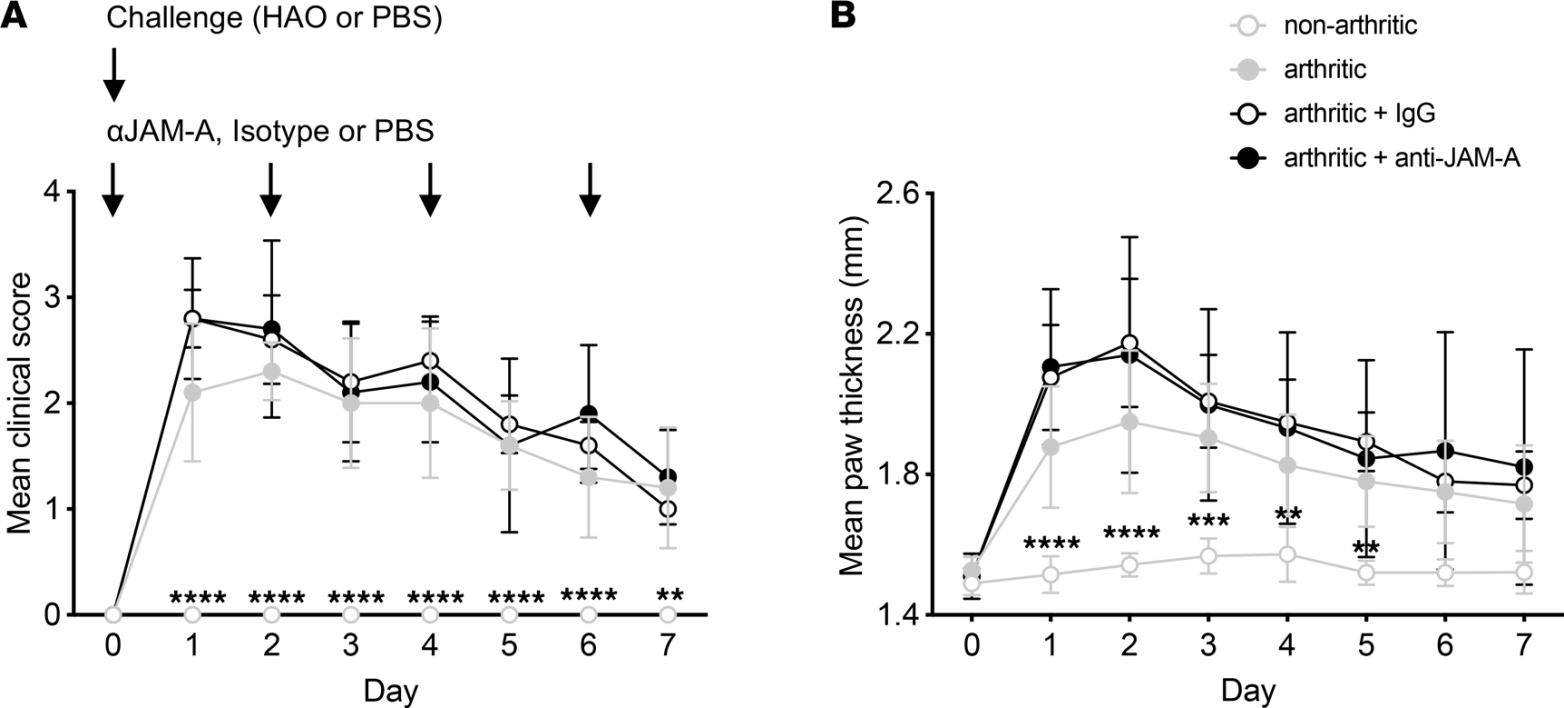
JAM-A blockade does not alter the development of arthritis. Inflammatory arthritis was induced in WT female C57BL/6 mice. Mice were treated with PBS, isotype control IgG, or anti–JAM-A i.p. shortly prior to HAO or PBS challenge and on days 2, 4, and 6 after challenge. The development of arthritis was monitored daily. (**A**) Mean hind paw clinical arthritis score. (**B**) Mean hind paw thickness. Arthritic groups *n* = 5; nonarthritic *n* = 4. Statistical differences between the treatment groups and the arthritic + IgG group using 2-way repeated-measures ANOVA with Dunnett’s multiple-comparison test; **P < 0.01, ***P ≤ 0.001, ****P ≤ 0.0001. Data are from a single experiment and show the mean ± SD.

In order to evaluate the potential of JAM-A as a therapeutic target in arthritis, mice were treated during the induction phase (days 0, 2, 4, and 6 after HAO injection) with a blocking anti–JAM-A antibody (14). Signs of clinical arthritis and marked swelling developed in all groups administered HAO. Treatment with anti–JAM-A, however, did not alter the development or progression of disease (Figure 6, A and B).

**Figure 7:**
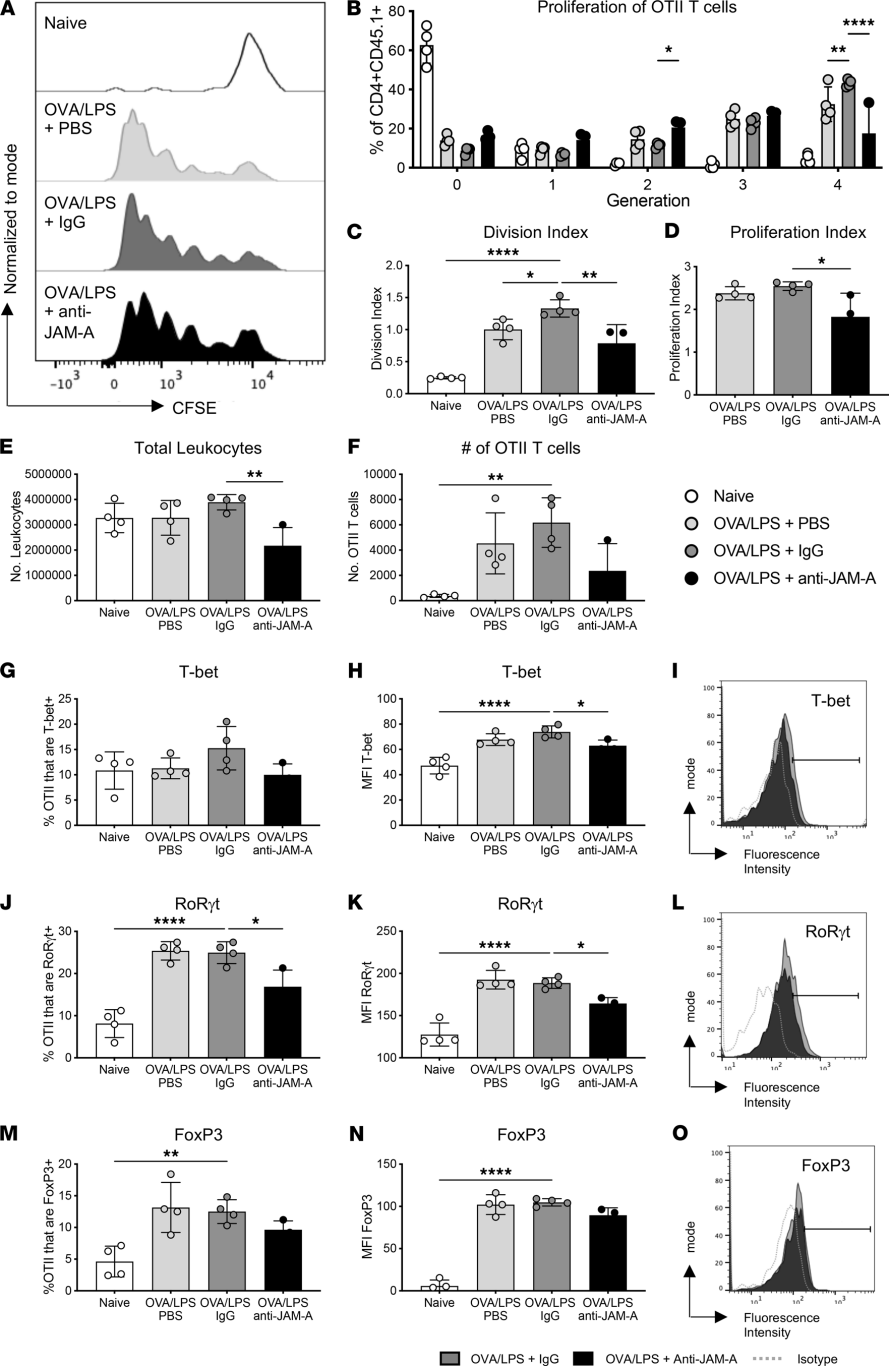
JAM-A blockade in vivo attenuates CD4^+^ T cell proliferation and decreases T-bet and RORγt expression by recently primed T cells. CFSE-labeled immune cells from LNs and spleens of OT-II mice were adoptively transferred to female C57BL/6 mice that were challenged with footpad injections of LPS and 0.5 ug OVA in the presence of anti–JAM-A mAb or its IgG isotype control. Popliteal LNs were harvested 72 hours later and analyzed by flow cytometry. (**A**) Representative histograms of CFSE fluorescence intensity on OT-II CD4^+^ cells (CD4^+^CD45.1^+^) with gating on cell generations and (**B**) quantification of the proportion of cells in each generation. (**C**) Division index and (**D**) proliferation index of OT-II CD4^+^ T cells. (**E**) The total number of leukocytes in the draining LNs. (**F**) The total number of OT-II CD4^+^ T cells in the draining LNs. (**G**) Percentage T-bet^+^. (**H**) MFI of T-bet. (**I**) Representative histograms showing T-bet expression on OT-II CD4^+^ T cells. (**J**) Percentage RORγt^+^. (**K**) MFI of RORγt. (**L**) Representative histograms showing RORγt expression on OT-II CD4^+^ T cells. (**M**) Percentage FoxP3^+^. (**N**) MFI of FoxP3. (**O**) Representative histograms showing FoxP3 expression on OT-II CD4^+^ T cells. Data are from a single experiment; each symbol represents an individual animal; mean ± SD shown; *n* = 4. Statistical differences between the treatment groups and the OVA/LPS IgG-treated control were determined using 1-way ANOVA with Dunnett’s multiple-comparison test. *P ≤ 0.05, **P ≤ 0.01, ****P ≤ 0.0001.

The majority of the JAM-A^+^ cells migrating to the joint during inflammation (~70%) were identified as cDC2 (Figure 5C), a cell type with a role in T cell activation. Additionally, our previous studies identified a role for JAM-A in the development of T cell–DC interactions, a critical step in T cell activation (15). We therefore assessed the effect of blocking JAM-A during T cell priming in vivo using an adoptive transfer model (Supplemental Figure 7). The effect of JAM-A blockade on T cell proliferation, activation, and differentiation in this model would therefore provide a direct measure of the role of JAM-A in these important cellular interactions. Proliferation of OT-II T cells from pLNs was assessed by measuring CFSE dilution with the percentage of cells at generations 0–4 determined and the division and proliferation indexes calculated. The division index, defined as the mean number of divisions all cells have undergone, shows the response of the whole population, while the proliferation index is the mean number of divisions that the dividing cells have undergone and measures the division of the responding population. Treatment with anti–JAM-A limited proliferation with an increased percentage of cells in division 2, a reduction in the percentage of cells in division 4, and a reduction in division and proliferation indexes compared with the isotype treatment (Figure 7, A–D). The total number of cells in the LNs of the JAM-A–treated mice was significantly lower than the IgG isotype–treated animals (Figure 7E); the number of OT-II T cells in the LNs, although lower, was not significantly different (Figure 7F). The activation of the OT-II T cells assessed by expression of CD44 and CD62L was not significantly altered by anti–JAM-A (data not shown). Blockade of JAM-A reduced the expression of both T-bet (MFI only) (Figure 7, G–I) and RORγt (percentage and MFI) (Figure 7, J–L), whereas the expression of FoxP3 was unaffected (Figure 7, M–O). These data suggest that JAM-A plays a role in the differentiation of CD4^+^ T cells.

## Discussion

This study aims to understand the genetic signals that govern the retention of cells within the inflamed joint, a vital and often overlooked aspect of tissue inflammation. Using the Kaede photoconvertible system, we identified cells that migrated from the inflamed tissue to the draining LNs and those that remained in the inflamed tissue. Comparison of these 2 cell populations by next-generation sequencing produced a list of genes whose expression was differentially regulated between these populations. Many of the genes identified have known roles during inflammatory processes, including the gene that encodes S1PR1. We demonstrated that inhibition of the S1PR1/S1P1 pathway reduced CD4^+^ T cell egress from the inflamed joint, validating the approach used to identify genes with roles in retention and egress from sites of inflammation. We refined the gene expression analysis by comparing transcript analysis from inflamed skin, thereby identifying genes whose regulation was altered specifically in joint inflammation. Further refining this list to genes with roles in cell adhesion and migration, we identified 15 genes upregulated in cells retained specifically in the inflamed joint and further characterized the role of 1 molecule, JAM-A. We demonstrated that although blocking the activity of this molecule did not alter the development of joint pathology, it did affect the proliferation and differentiation of naive T cells in vivo.

Using the transgenic Kaede mouse allowed us to reliably identify cells that had moved out of the inflamed joint versus those that were retained, and analysis of the transcriptome of these cells identified several differentially expressed genes that have previously been associated with disease in arthritis models and in RA. For example, genes such as *Ccr1* and *Ccr2* with known roles in the recruitment of inflammatory cells to sites of inflammation were upregulated in the cells retained in the joints. The expression of CCR1 has been shown to be increased in synovial tissue of patients with RA (16–18) and in animal models of arthritis (19); deficiency of CCR1 has been shown in animal models to limit the development of arthritis and other experimental models of inflammatory disease, but blockade of CCR1 has been trialed in RA with only limited success (20, 21). Genes upregulated in the cells that had migrated were those involved in LN trafficking, such as *Cxcr3* and *S1pr1*. A role for CXCR3 in the accumulation of cells in LNs but not in the inflamed joints has been demonstrated in mice (22), which is consistent with our data showing an increased expression on the migrated cells, further validating our approach.

Our data also identified the upregulation of S1PR1 in migrated cells compared with nonmigrated cells. S1PR1 is 1 of 5 receptors for S1P, which plays a key role in the migration of lymphocytes out of LNs, allowing the migration of cells expressing S1PR1 from areas of low S1P (LNs) to regions with higher S1P concentrations, such as the blood. The inhibition of S1PR1 using the functional antagonist FTY720 not only results in the retention of T cells within LNs but also has been shown inhibit the persistence of CD4^+^ T cells in inflamed skin (23). FTY720 treatment has also been shown to attenuate the development of arthritis in the collagen-induced arthritis model and to prevent the migration of DCs to the LNs (8). By contrast, in our model, we demonstrated that the inhibition of S1PR1 by FTY720 reduced CD4^+^ T cell migration to the LNs but did not alter DC migration, which may reflect differences in the treatment regimen utilized in the 2 studies. In our model, we only treated with FTY720 at the point of photoconversion, whereas Han et al. treated animals for the duration of the CIA model (8).

The identification of genes known to play a role in cell migration and inflammation in our analysis, combined with our demonstration of the impact of direct blockade of one of these genes on cell accumulation, validated our approach for finding disease-associated genes. However, a number of genes identified in this analysis are broadly applicable to a range of inflammatory conditions, and blocking these pathways may lead to significant immunosuppression, leaving individuals vulnerable to infection or cancers. The ability to target inflammation in a tissue- or disease-specific fashion offers the potential to provide improved therapeutics with fewer side effects. We therefore induced inflammation in a non-joint tissue site (the skin) and by comparing the gene expression patterns between the joint and skin retained and migrated cells, the list was further refined, identifying genes that were differentially regulated specifically in joint inflammation. Within these genes, analysis of pathways associated with cell migration and adhesion identified 15 genes that were upregulated specifically in the joint-retained cells. Of course, we note the caveat that differentially expressed genes from the RNA-Seq data may also be a reflection of distinct cell types in these populations rather than cell type–independent molecular mechanisms of cellular trafficking.

From this shortlist of joint-specific genes, we selected JAM-A for further study. JAM-A is expressed by platelets and endothelial, epithelial, and immune cells such as monocytes and DCs (14, 24). Ligands for JAM-A include itself in the form of homophilic interactions, other JAM family molecules, LFA-1, and CD9 (14, 25–27). JAM-A plays important roles in endothelial cell migration and barrier function. In vitro blockade inhibits the transmigration of monocytes, neutrophils, and T cells, while JAM-A deficiency in mice has been shown to increase DC trafficking to LNs and to increase contact hypersensitivity responses (24). JAM-A has been shown to play complex roles in a variety of inflammatory conditions reviewed in Bonilha et al. (28). *F11r* was reported to be upregulated in PBMCs of patients with RA (29). In addition, JAM-A was shown to be expressed in inflamed joints of K/BxN mice (29), animals expressing transgenic TCR and MHCII that develop severe inflammatory arthritis.

In a model of RA driven by autoantibodies, treatment with anti–JAM-A mAb delayed the disease onset and partially ameliorated overall disease, similar to the effect observed after treatment with anti-ICAM1, another LFA-1 ligand (29). These data suggest that the therapeutic effects in this model may result from disruption of LFA-1 pathways, with treatment using a mAb targeting the α chain of LFA-1 promoting greater amelioration of clinical disease. The inflammation in the serum-transfer model is initiated by autoantibodies, while our inflammatory arthritis model was mediated by antigen-specific Th1 cells, ultimately leading to adaptive responses against self-antigens. We showed that blockade of JAM-A during the disease induction phase in the arthritis model did not alter the development of arthritis. However, blockade during priming limited T cell proliferation and differentiation to Th17 and Th1 phenotypes. It has previously been noted that JAM-A may enhance stable interactions between T cells and APCs. In the context of T cell activation, JAM-A interaction with LFA-1 may be of particular importance because this molecule is expressed on T cells, forming a part of the immunological synapse. Our previous work has demonstrated that JAM-A is present within the immunological synapse and is therefore available to bind and form part of the interaction (15). These data suggest that although the blockade of JAM-A during the acute induction phase of our arthritis model was insufficient to alter disease progression, it may play a role in the initiation of immune responses and, as such, warrants further investigation. Overall, our study demonstrated that the molecules involved in cell egress from or retention in inflamed tissues are potentially important targets for the development of therapeutics in autoimmune disease.

Using a combination of cell-tracking approaches and murine models of inflammatory arthritis, we have identified genes and pathways regulating cell migration in a variety of inflamed sites and anatomically specific tissue signatures. This skin- and joint-specific data set will be an invaluable resource for the identification of therapeutic targets for arthritis and other inflammatory disorders.

## Methods

### Mice.

OVA-specific OT-II CD45.1 mice on a C57BL/6 background (14) were bred in-house. C57BL/6 mice were obtained from Envigo (product code 057). Kaede-transgenic mice provided by Michio Tomura (Osaka Ohtani University, Osaka, Japan) and Osami Kanagawa (University of Lyon, Lyon, France) (11) were bred in-house. OT-II CD45.1 mice were used as donors and Kaede-transgenic or C57BL/6 mice as recipients. Age- and sex-matched mice aged between 6 and 10 weeks were used for all experiments. All animals were specific pathogen free and housed in standard animal house conditions at the University of Glasgow in accordance with UK Home Office Regulations.

### Induction of inflammatory arthritis and ear pinna inflammation.

CD4^+^ T cells from OT-II CD45.1 mice were polarized in vitro over 72 hours toward a Th1 phenotype in the presence of 1 μg/mL peptide OVA_323-339_ (pOVA) (Sigma-Aldrich), 10 ng/mL recombinant IL-12, and 2 μg/mL anti–IL-4 (both R&D Systems). Recipient mice received 3 × 10^6^ in vitro–polarized Vα2^+^Vβ5^+^ OT-II Th1 cells (i.v.). The following day, mice were immunized with 100 μg OVA (Worthington Biochemical) in CFA (Sigma-Aldrich) in the scruff of the neck. Ten days later, mice were challenged in the footpad with PBS (Gibco) or 100 μg HAO as previously described (10). Ear pinna inflammation was induced following the same protocol, except that the mice were challenged with either PBS or 100 μg HAO in the ear pinna.

For the JAM-A blocking studies, mice received (i.p.) 100 μg of anti–JAM-A mAb BV11 (Sigma-Aldrich) or Ultra-LEAF Purified Rat IgG2b mAb RTK4530 (isotype control; BioLegend) on days 0, 2, 4, and 6 after challenge.

### Photoconversion of Kaede tissue.

Photoconversion of Kaede mouse tissues was performed using a 12× S06J Blu-ray diode with a 405-G-2-glass lens (DTR’s Laser Shop) emitting at 405 nm with mean power of 600–650 mW. For joints, the ventral and dorsal aspects of the intact hind paw were exposed to UV light for three 5-second bursts with 3-second intervals (30). Ear pinna photoconversion was as for joints but using three 3-second bursts of UV exposure. The area around the target tissues was shielded from UV exposure with black fabric. Tissues were photoconverted on day 3 after challenge. Inflamed tissues and draining LNs were harvested 24 hours later.

### Recovery of cells from inflamed tissues and LNs.

Ankle joints or ear pinnae were teased apart using forceps; excised LNs were passed through Nitex mesh (Cadisch Precision Meshes) and both tissues digested for 25 minutes at 37°C with 2.64 mg/mL collagenase D (Roche). Joint tissue was then homogenized using the Miltenyi Biotec Tissue Dissociator and passed through a 70 μm cell strainer to obtain a single-cell suspension. Joint and LN single-cell suspensions were then washed in FACS buffer (PBS/2% FCS/2 mM EDTA) and stained for flow cytometry analysis.

### Antibodies, flow cytometry, and cell sorting.

Cells were stained for viability in PBS using fixable viability dye (eFluor506; eBioscience) for 30 minutes at 4°C. Cells were then washed in FACS buffer and subsequently incubated with Fc receptor block for 10–15 minutes at 4°C, and then appropriate fluorochrome-conjugated antibodies (Supplemental Table 7) were added and the cells stained for 30 minutes at 4°C. For transcription factor staining, the transcription factor buffer set (BD Pharmingen) was used as per the manufacturer’s instructions. All cells were then washed in FACS buffer, resuspended in FACS buffer, and acquired on an LSR II (BD Biosciences). Data were analyzed using FlowJo software (v10.2). For generating t-SNE plots, events were first downsized and biological replicates were concatenated. Viable single CD45^+^Kaede red^+^ cells were sorted using an Aria III (BD Biosciences). Sorted cells were subsequently washed twice in ice-cold PBS and lysed in RLT buffer containing 1% β-mercaptoethanol and stored at –80°C until further analysis.

### RNA-Seq analysis.

TruSeq next-generation sequencing was performed on a HiSeq 2000, pair ended with a 100 bp read length (Source Bioscience). For data processing and alignment, a high read quality (mean score above 28) in all samples was confirmed using FastQC. To remove adapters, ensure proper read pairing, and trim reads with low start or end read quality, the package Trim-Galore! was used under default settings. Next, reads were aligned to the mm10 genome using TopHat2, with the flags --b2-sensitive, --no-coverage-search, and version 87 of the mouse transcriptome. Fragments per kilobase of exon per million mapped fragments were generated using Cufflinks under default settings. All samples had a concordant pair alignment rate above 75% and at least 20 million aligned concordant read pairs. Read counts were generated using HTSeq count under default settings. Differential expression was called using the Bioconductor package DESeq2 with adjusted *P* less than 0.05 and absolute log_2_fold greater than 1 considered significant. The data were visualized using Searchlight2 and R (31).

### Gene ontology enrichment.

To determine enrichment of gene ontologies, we used a standard hypergeometric test with Benjamini-Hochberg multisample correction and adjusted *P* value threshold of less than 0.05 for significance. The gene ontology database was selected as a combination of the Molecular Signatures Database (http://software.broadinstitute.org/gsea/msigdb/collections.jsp) collections: canonical pathways, BIOCARTA, KEGG, REACTOME, and GO. The data are available in National Center for Biotechnology Information’s Gene Expression Omnibus (accession GSE195817).

### Adoptive transfer model.

Adoptive transfer was performed as previously described (32). Briefly, cell suspensions obtained from spleens and LNs of OT-II mice were stained with 1 μM CFSE (Thermo Fisher Scientific). Next, 1 × 10^6^ CD4^+^ OT-II T cells were transferred (i.v.) into C57BL/6J recipients. One day later, mice received (i.p.) 100 μg of anti–JAM-A mAb BV11 (Sigma-Aldrich) or Ultra-LEAF Purified Rat IgG2b mAb RTK4530 (BioLegend). After the antibody administration, mice were immunized with 0.5 μg OVA (Worthington Biochemical)/8 μg LPS (Sigma-Aldrich) or LPS alone in the hind footpads. Three days later, pLNs were harvested for flow cytometry analysis.

### Clinical assessment of arthritis.

Immediately prior to and daily after HAO challenge, mice were monitored for signs of arthritis. Hind (challenged) paws were assessed following a 0–4 scale of ascending severity as previously described (33). Paw thickness was determined in millimeters using digital calipers (Kroeplin). Data are presented as the mean paw score and paw thickness.

### Statistics.

Data are shown as mean ± SD. Statistical significance was determined using 2-tailed Student’s *t* test and 1-way or 2-way ANOVA as appropriate. Specific tests and significance levels are indicated in the appropriate figure legends. A *P* value of less than 0.05 was considered statistically significant. Statistical analysis of results was performed using GraphPad Prism versions 6 and 8.

### Study approval.

All animal experiments were performed under a UK Home Office license in accordance with UK Home Office regulations following approval by the University of Glasgow Ethics Committee.

## Author contributions

CTP, RAB, JMB, and PG conceived and designed the study. CTP, RAB, HES, and CSB acquired data. CTP, RAB, HES, CSB, and JJC analyzed and interpreted data. CTP, RAB, HES, CSB, JJC, IM, JMB, and PG wrote, reviewed, and/or revised the manuscript. CTP and RAB contributed equally to the manuscript. HES and CSB contributed equally to the manuscript. The authorship order among co–first and co–second authors was agreed upon after discussion by the authors.

## Supplementary Material

Supplemental data

Supplemental tables 1-7

## Version 1 02/22/2022

In-Press Preview

## Version 2 04/08/2022

Electronic publication
