# Supplementary material for: Dissecting the molecular control of immune cell accumulation in the inflamed joint
Source: JCI Insight. 2022 Apr 8;7(7):e151281. doi: 10.1172/jci.insight.151281 (PMC9057592; doi:10.1172/jci.insight.151281)
Supplement: Supplemental data [file jciinsight-7-151281-s086.pdf]

SUPPLEMENTAL FIGURES & LEGENDS

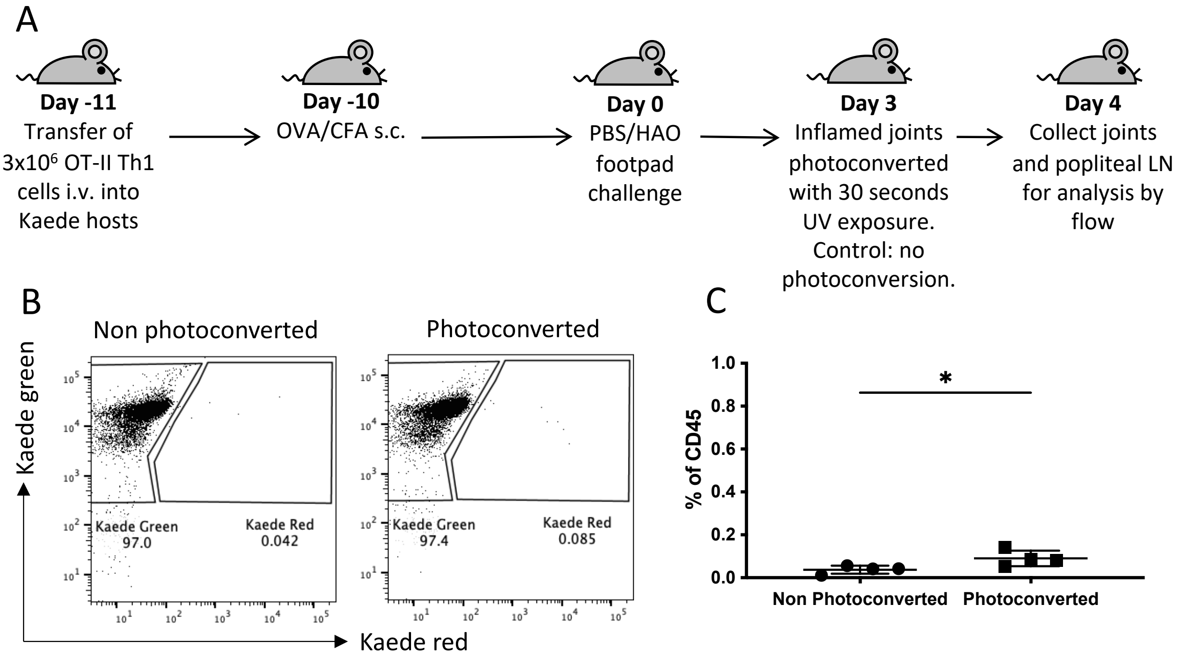

Supplementary Figure 1. Inflammatory arthritis model in Kaede mice.

(A) Schematic showing the OVA-RA model timeline using transgenic Kaede mice as the recipient mice. On day -11, 3 x 10^6 Th1 polarized CD4+ OTII T cells were transferred into transgenic Kaede mice, the following day mice were immunized in the scruff with 100 µg OVA in CFA. On day 0 mice were challenged with HAO in the footpad. On day 3 post challenge footpads were photoconverted by exposure to a total of 30 seconds of UV light. Twenty-four hours later mice were sacrificed, and the popliteal lymph nodes collected for analysis via flow cytometry.

The extent of incidental photoconversion of the popliteal lymph nodes determined by flow cytometry (B) plots showing $CD45^+$ cells highlighting Kaede green versus Kaede red in non-photoconverted and photoconverted HAO challenged mice recovered from the popliteal lymph nodes harvested shortly after photoconversion. Shown as a percentage of $CD45^+$ gate. (C) Quantitative analysis of extent of photoconversion showing the percentage of Kaede red cells. Statistical analysis: Unpaired t test; * represents $p<0.05$; n=4.

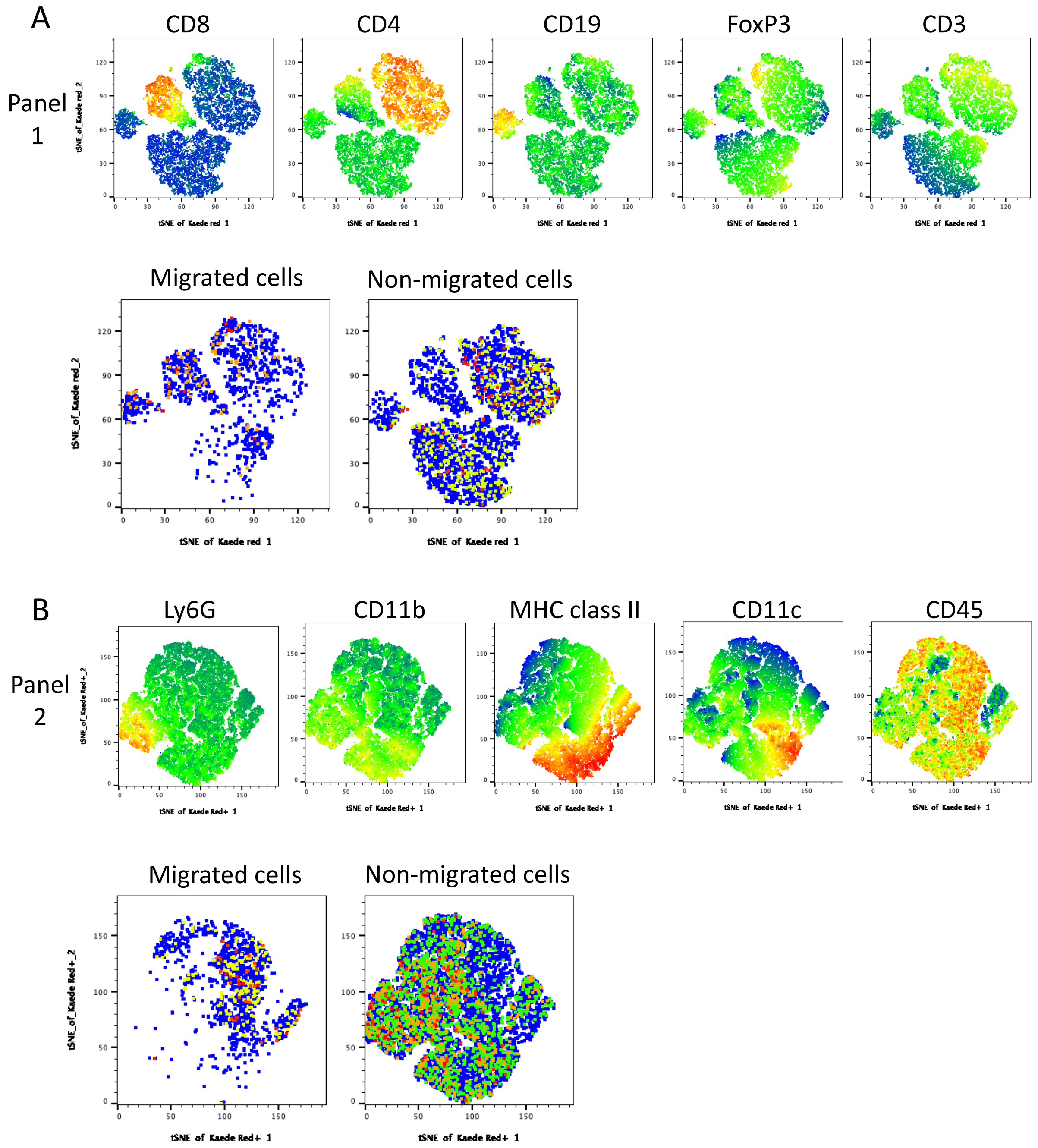

Supplementary Figure 2. Non-migrated cells in the inflamed joint and migrated cells in the draining lymph nodes are comprised of distinct cell groups; t-SNE analysis. (A) Representative t-SNE plots showing migrated and non-migrated cells stained for CD8, CD4, CD19, FoxP3 and CD3. (B) Representative t-SNE plots showing migrated and non-migrated cells stained for Ly6G, CD11b, MHC-II, CD11c and CD45.

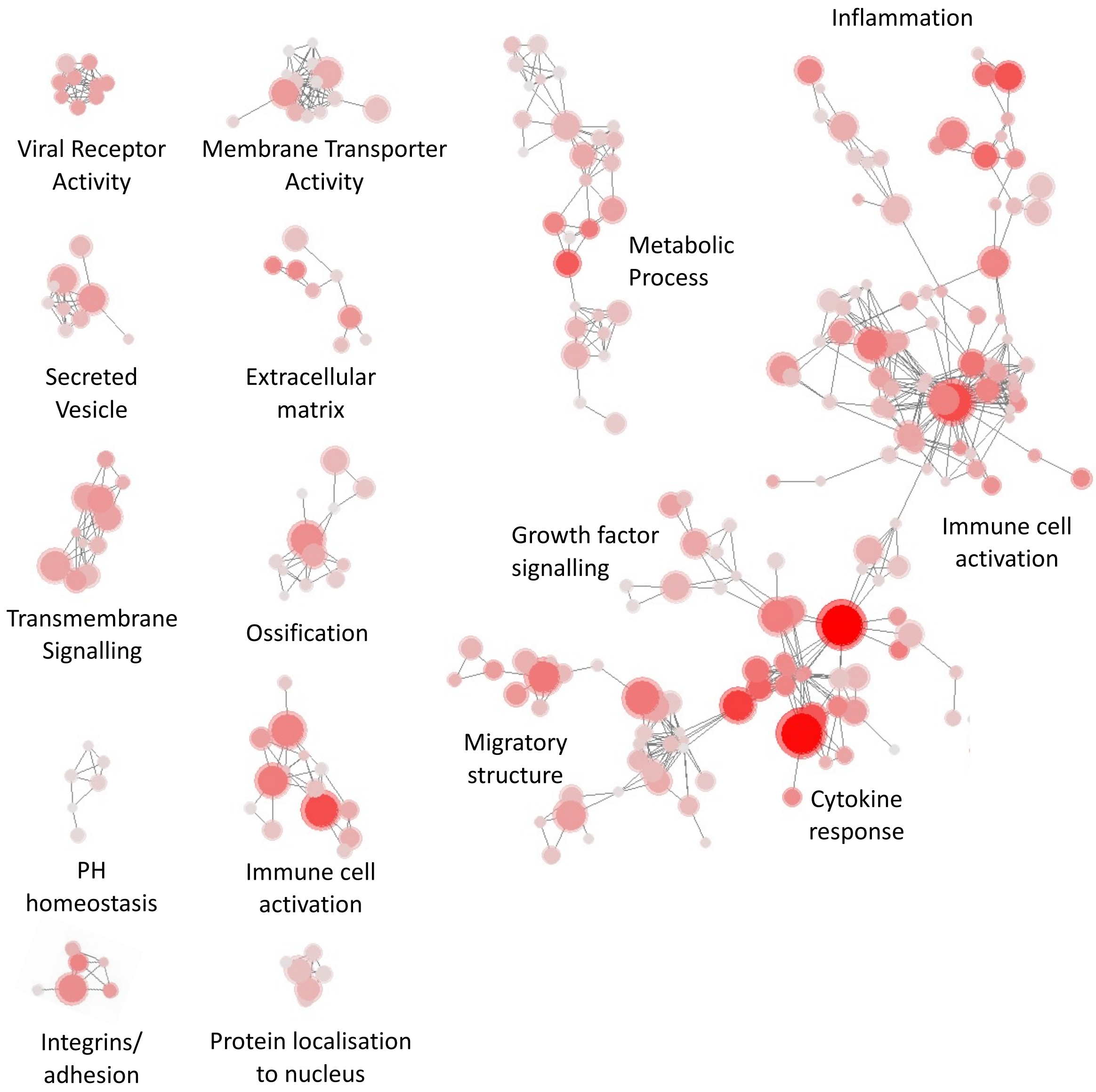

Supplementary Figure 3: Network analysis of the upregulated genes in non-migrated cells. Network analysis showing genes upregulated in the non-migrated (joint) Kaede red cells was performed and filtered for clusters containing 6 or more differentially expressed genes. Clusters were named based on the function of the genes within them.

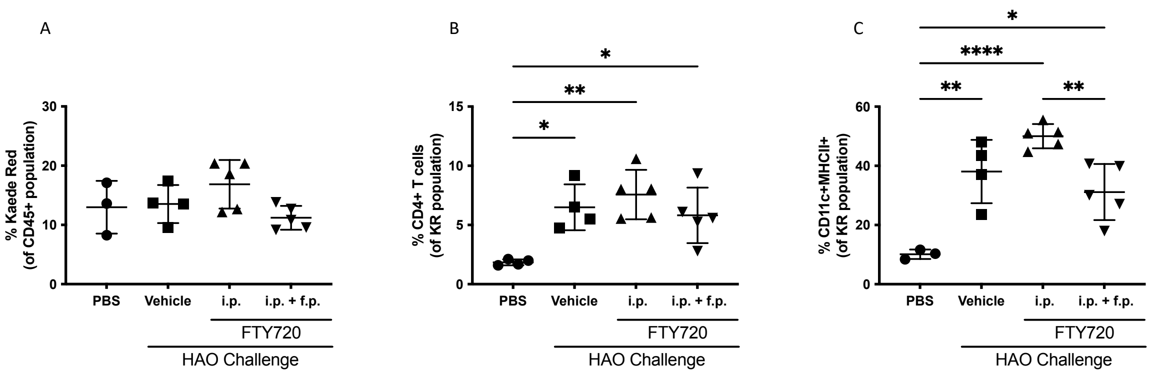

Supplemental Figure 4. Blockade of the S1P pathway via FTY720 administration, affects the movement of cells out of the inflamed joint. (A) Summary of flow cytometric analysis showing the percentage of Kaede Red non-migrated cells in the joint after PBS or HAO footpad challenge followed by vehicle or FTY720 administration. Previously gated on the $CD45^+$ cells. Flow cytometric analysis of the Kaede Red population specifically shows (B) the percentage of the Kaede Red population that are $CD3^+CD4^+$ T cells and (C) the percentage of Kaede Red cells that are $CD11c^+MHC\text{-}II^+$. Statistical differences between the treatment groups were determined using one way ANOVA with Tukey multiple comparisons test. Data is representative of two separate experiments; each symbol represents and individual animal and shows the mean $\pm$ SD. * represents $p<0.05$, ** represents $p<0.01$, **** represents $p<0.1$.

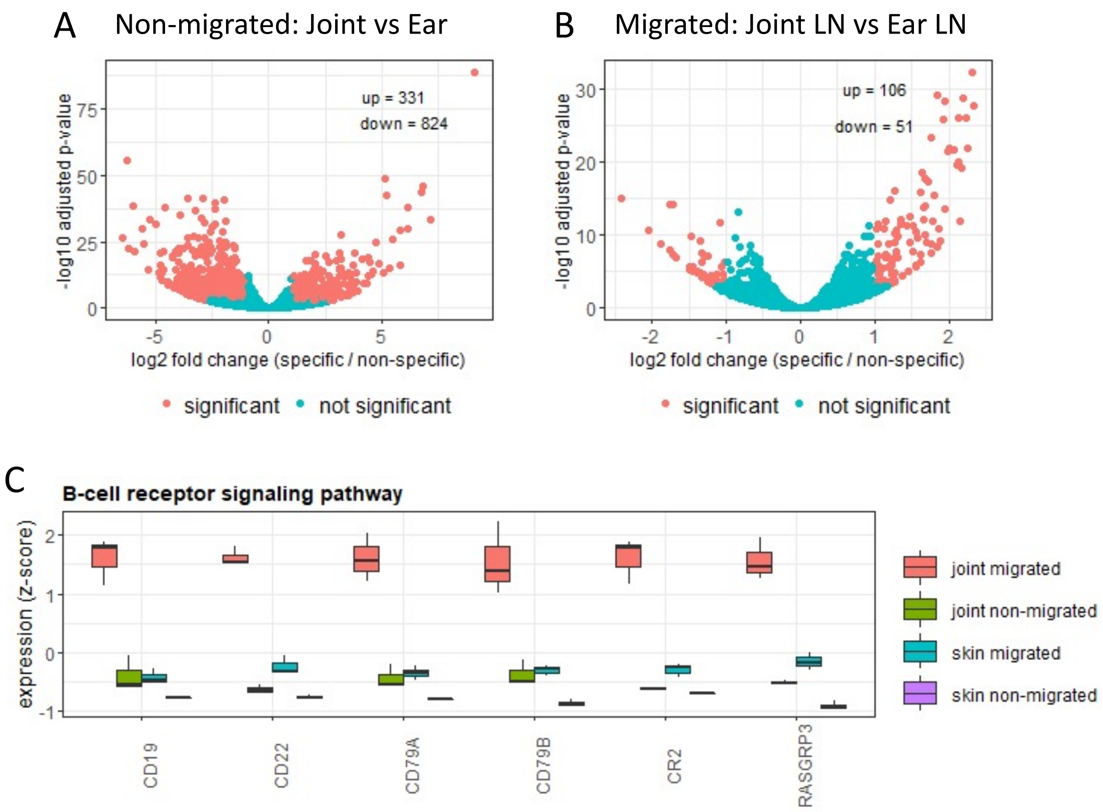

Supplemental Figure 5: Joint vs Skin volcano plots and B cell signaling genes

Volcano plots highlighting genes that are more than two-fold up- or down-regulated between (A) the joint vs ear non-migrated populations [331 genes up-regulated; 824 genes down-regulated] and (B) the joint vs ear migrated populations [106 genes up-regulated; 51 genes down-regulated]. (C) Box and whisker plots showing the relative expression of each of the genes involved in the 'B cell receptor signaling' pathway in the joint migrated, joint non-migrated, skin migrated and skin non-migrated groups. The box represents the mean, lower and upper hinges correspond to the 25th and 75th percentiles and the whiskers represent the maximum and minimum.

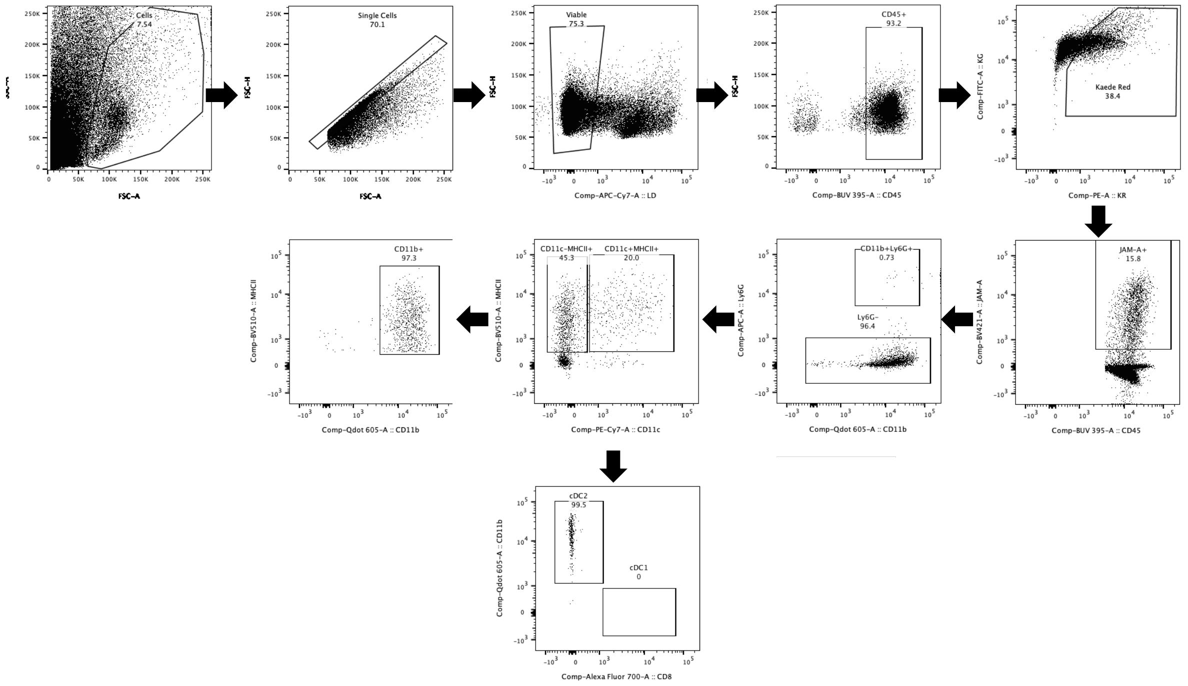

Supplemental Figure 6. Gating strategy used to phenotype the Kaede red JAM-A+ cells in the joint and LN. Inflammatory arthritis was induced in Kaede mice, tissues where photoswitched at 3 days post HAO challenge and 24 hours later the immune cells were analysed by flow cytometry. Leukocytes were identified by size and granularity; doublets and dead cells excluded and CD45+ cells gated. Migrated and non-migrated cells were identified as Kaede red. JAM-A expressing Kaede red cells were identified and subsequently gated to identify neutrophils (CD11b+Ly6G+), Macrophages/Monocytes (CD11c-CD11b+MHCII+), cDC1 (CD11c+MHCII+CD8+) and cDC2 (CD11c+MHCII+CD11b+).

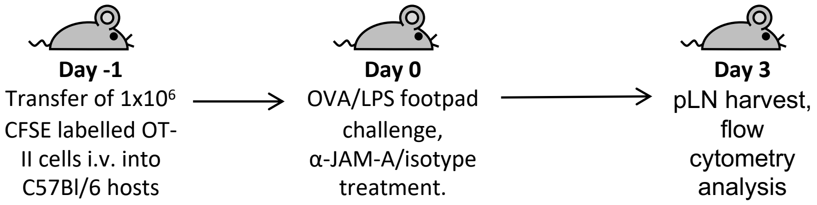

Supplemental Figure 7. Schematic of adoptive transfer model and anti-JAM-A/isotype treatment. (A) Schematic showing the adoptive transfer timeline using C57Bl/6 mice as the recipient mice. On day -1 mice received CFSE labelled immune cells containing 1 x 10^6 CD4+ OTII T cells. On day 0 mice were immunised in the footpad with 0.5 μg OVA/8 μg LPS or LPS alone and were treated with anti-JAM-A mAb, the isotype control or PBS I.P. Popliteal LNs were harvested 72 hours later and analyzed by flow cytometry.
